# Identification of a novel bovine copiparvovirus in pooled fetal bovine serum

**DOI:** 10.1007/s11262-020-01757-1

**Published:** 2020-04-18

**Authors:** Sally A. Baylis, Csaba Miskey, Johannes Blümel, Marco Kaiser, Beatrix Kapusinszky, Eric Delwart

**Affiliations:** 1 Paul-Ehrlich-Institut, Langen, Germany; 2 GenExpress Gesellschaft für Proteindesign mbH, Berlin, Germany; 3 Vitalant Research Institute, San Francisco, CA 94118 USA; 4 Department of Laboratory Medicine, University of California San Francisco, San Francisco, CA 94118 USA

**Keywords:** Parvovirus, *Copiparvovirus*, Bovine sera

## Abstract

A novel parvovirus was identified as a cell culture contaminant by metagenomic analysis. Droplet digital PCR (ddPCR) was used to determine viral loads in the cell culture supernatant and further analysis, by ddPCR and DNA sequencing, demonstrated that fetal bovine serum (FBS) used during cell culture was the source of the parvovirus contamination. The FBS contained ~ 50,000 copies of the novel parvovirus DNA per ml of serum. The viral DNA was resistant to DNAse digestion. Near-full length sequence of the novel parvovirus was determined. Phylogenetic analysis demonstrated that virus belongs to the *Copiparvovirus* genus, being most closely related to bovine parvovirus 2 (BPV2) with 41% identity with the non-structural protein NS1 and 47% identity with the virus capsid protein of BPV2. A screen of individual and pooled bovine sera identified a closely related variant of the novel virus in a second serum pool. For classification purposes, the novel virus has been designated bovine copiparvovirus species 3 isolate JB9 (bocopivirus 3-JB9).

**Electronic supplementary material:**

The online version of this article (10.1007/s11262-020-01757-1) contains supplementary material, which is available to authorized users.

## Introduction

Parvoviruses are small non-enveloped viruses with single-stranded DNA genomes ~ 5 to 6 kb in length and possess two main open reading frames (ORFs) encoding non-structural proteins involved in transcription and DNA replication as well as the capsid protein(s) [1]. They belong to the *Parvoviridae* family of viruses which includes the subfamilies *Parvovirinae* infecting vertebrates and the *Desovirinae* that infect arthropods [1]. The *Parvovirinae* contain eight different genera: *Amdoparvovirus*, *Aveparvovirus*, *Bocaparvovirus*, *Copiparvovirus*, *Dependoparvovirus*, *Erythroparvovirus*, *Protoparvovirus* and *Tetraparvovirus*. With the increase in metagenomic studies, the number of parvovirus species, from a diverse range of hosts, has greatly increased in recent years [2–4] and transcriptome studies suggest that parvoviruses might span the entire animal kingdom [5].

The genus *Copiparvovirus* contains two species recognized by the International Committee on the Taxonomy of Viruses (ICTV), the first of which *Ungulate copiparvovirus 1* (bovine parvovirus 2 or BPV2) was identified in 2001 in commercial bovine serum [6]. *Ungulate copiparvovirus 2* (porcine parvovirus 4) was found in the lung lavage of a diseased pig, co-infected with porcine circovirus type 2 in the USA in 2005 [7]. Further, related, but unclassified viruses have since been reported including two viruses identified in pigs—porcine parvovirus 5 [8] and porcine parvovirus 6 [9] and a virus termed Sesavirus found in the feces of a Californian sea lion pup [10]. During an investigation of the bovine virome of US calf serum, bosavirus was identified [3] and has been found in cattle persistently infected with pestiviruses [11]. A virus identified in sera from European roe deer has been designated *Ungulate copiparvovirus 3* and this virus was first detected in *Ioxodes ricinus* ticks found on the deer [12]. Horse parvovirus CSF was found in the cerebrospinal fluid of an animal with neurological deficits [2] and a second equine virus, termed EqPV-H, was detected in the liver of a horse that died of equine serum hepatitis—Theiler’s disease [13]. Further studies with EqPV-H have shown a strong association with cases of fatal Theiler’s disease and subclinical hepatitis in animals in contact with the animals where a fatal outcome was observed [14]. The study we present here describes the identification of a novel bovine copiparvovirus, designated bovine copiparvovirus species 3 isolate JB9 (bocopivirus 3-JB9) and a related virus, MK1 that shares 99% nucleotide identity with JB9, found in pooled fetal bovine serum (FBS).

## Results

Cell culture supernatant from A549 cells infected with hepatitis E virus (HEV) obtained from swine feces was investigated by metagenomic analysis as previously described [15]. Sequences were compared to the GenBank non-redundant protein database using BLASTx with an *E* value cutoff of < 10^–5^. As expected, HEV sequences were obtained; however, a small number of reads were related to BPV2, a member of the *Copiparvovirus* genus, generating three discrete contigs: one mapping to the non-structural protein and the others to the viral capsid protein. Using PCR, it was demonstrated that the novel sequences were present in the FBS used during the HEV cell culture. Sequence gaps between the respective contigs were determined by PCR and sequencing using primers located near the ends of the contigs. The approach to extend the sequences at the 5′ and 3′ ends is described in the supplementary methods using a mutant Taq polymerase (SD polymerase) with high strand-displacement activity [16]. From the analysis of the 5′ end of the genome, it can be inferred, based on primer binding and extension, that positive sense viral genomes are packaged into virus particles.

Droplet digital PCR (ddPCR) was performed using specific primers and hydrolysis probes for the three contigs identified in the initial metagenomic analysis (Supplementary materials and methods). The ddPCR analysis revealed that the concentration of viral DNA was ~ 50,000 copies/ml of FBS irrespective of which assay was used suggesting products were amplified from the same template. Pre-treatment of the FBS with DNAse prior to nucleic acid extraction did not affect the copy number demonstrating that the novel parvovirus DNA was encapsidated and protected from nuclease digestion. The copy number in the cell culture supernatant was tenfold lower than in the bovine serum; this lower concentration reflected the dilution of the serum in the cell culture medium. There was no evidence of replication of the novel parvovirus in the cell culture (either in the cells or cell supernatant) when monitored by ddPCR (data not shown).

Further lots of pooled bovine sera (*n* = 7 from 4 commercial suppliers sourced in South America and Australia) as well as individual bovine sera or whole blood (*n* = 43, sourced in Europe) were tested for the novel parvovirus by real-time PCR (Supplementary Materials and Methods) using the three sets of primers/probes used in the ddPCRs. Table 1 provides a summary of the observed cycle threshold (*C*_T_) values for the samples that yielded positive results in at least one of the PCRs. Of the individual serum and whole blood samples, one serum sample had *C*_T_ values of > 37 for all 3 PCRs, whilst 3 of the whole blood samples had *C*_T_ values of > 37 just for the C2-qPCR. It was not possible to perform sequence analysis on these samples, likely due to low viral loads. In the case of the serum pools, one pool was positive in only one PCR with a *C*_T_ value of > 39, another pool, however, was positive in all three assays with *C*_T_ values ranging from ~ 29 to 32 and sequencing (Supplementary Table 1) showed it to be closely related to the virus identified by metagenomic sequencing. The virus strain identified by metagenomic sequencing was termed JB9 and the related virus was termed MK1, both were South American in origin and share ~ 99% nucleotide identity.

**Table 1:** Testing of individual bovine serum and blood samples for the presence of the novel bovine parvovirus by real-time—*C*_T_ values of samples testing positive in at least one of more of the three screening PCRs

Sample type	Code	Origin	C1-qPCR^a^	C2-qPCR^a^	C3-qPCR^a^
Pooled fetal bovine serum	A	South America	39.4	–	–
	B	South America	30.8	29.5	32.0
	C	South America	33.7	31.7	33.3
Bovine serum	i	Europe	38.0	36.8	39.9
Bovine whole blood	ii	Europe	–	37.5	–
	iii	Europe	–	37.4	–
	iv	Europe	–	40.2	–

^a^The PCRs refer to the primers and probe combinations used in the ddPCR assays described in Materials and methods and correspond to the three original contigs identified by metagenomic analysis. Sample C refers to the original serum pool containing strain JB9. The limit of detection of the respective real-time PCRs is ≤ 10 copies per reaction. All other samples tested were negative

**Fig. 1:**
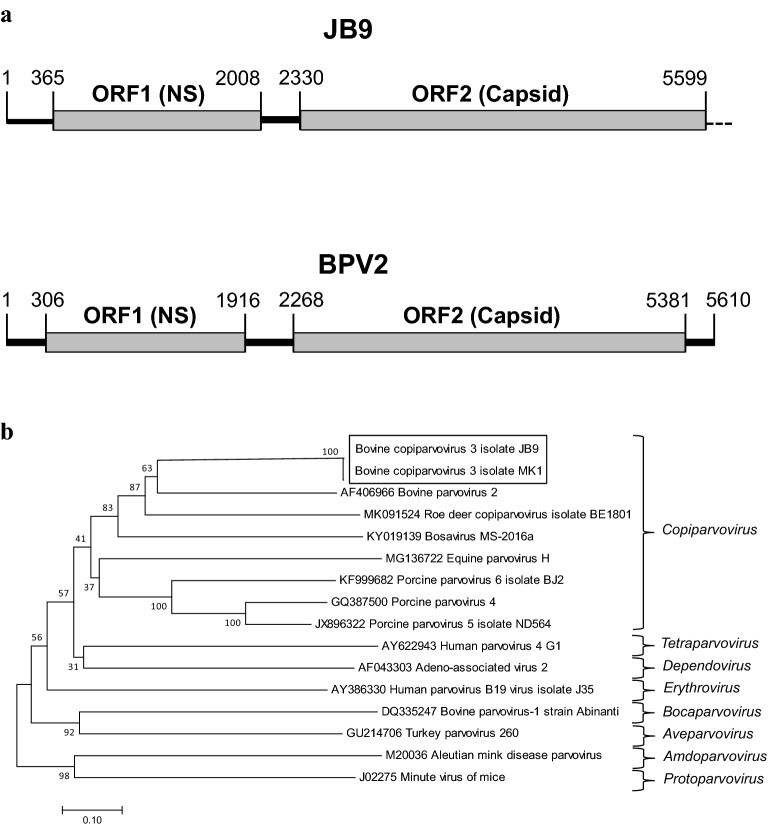
**a** Genome structure of bovine copiparvovirus 3 isolate JB9 (**a**) compared to bovine parvovirus 2 (Accession number AF406966). The single-stranded DNA genomes encode two open reading frames (ORFs), ORF 1 encoding the non-structural (NS) protein and ORF2 encoding the structural protein (capsid); the nucleotides numbers above each ORF indicate the position of the respective coding regions for the two viral sequences. The bovine copiparvovirus 3 isolate JB9 ORF2 is near-full length but incomplete. **b** Phylogenetic tree showing the relationship between bovine copiparvovirus 3 isolates JB9 and MK1and other parvoviruses. The analysis was performed using sequences of ORF1 and representative members of the *Copiparvovirus* genus and prototype strains of other *Parvovirinae* genera. Sequence alignment was performed using Clustal Omega (https://www.ebi.ac.uk/Tools/msa/clustalo/) with the default settings. A phylogenetic tree was generated using the neighbor-joining method based on the Kimura 2-paramter method in MEGA 7 [19]. The percentage of replicate trees in which the associated taxa clustered together in the bootstrap test (1000 replicates) are shown next to the branches

The sequence determined for strain JB9 is 5599 nucleotides in length with a GC content of ~ 44.5%. In the case of strain MK1, a sequence of 4743 nucleotides was determined. The two novel virus strains are ~ 99% identical and contain two large ORFs. In the case of strain JB9, ORF1 is 1644 nucleotides in length, encoding a protein of 547 amino acids and ORF2 is ~ 3273 nucleotides, encoding 1090 amino acids. It was not possible to amplify the sequences corresponding the very C-terminus of ORF2 of either strain. Comparison of the JB9 ORF2 amino acid sequence with that of BPV 2 reveals that the JB9-encoded sequence is actually slightly longer than that of BPV2, suggesting that the JB9 sequence is unlikely to be significantly longer and is close to being full length. The two main bovine parvovirus JB9 ORFs are separated by 322 nucleotides (as is the case with MK1), this is similar to BPV2 where the distance separating the two main ORFs is 352 nucleotides [6]. Both ORF1 and ORF2 are in the same reading frame and 36 nucleotides’ downstream from the ORF1 stop codon is a further small putative peptide of 45 amino acids, which is found in the same reading frame as both ORF1 and ORF2. The ORF1-encoded protein is homologous to the non-structural protein of parvoviruses NS1, showing greatest amino acid identity with BPV2 (40%); the organizational structure is shown in Fig. 1a. Similar to other parvoviruses, ORF1 contains a HXH domain (amino acids 94–96)—a metal-binding catalytic unit of the endonuclease domain, involved in parvovirus DNA replication as well as helicase motifs (i.e., GPXNTGKX—amino acids 331–338) and ATPase motifs involved in viral DNA replication [4]. The amino acid sequence of the ORF2-encoded protein is homologous to the main viral capsid protein (VP1) of parvoviruses, sharing ~ 47% amino acid identity with VP1 of BPV2 over the near-full length sequence of the strain JB9. The amino acid sequence of the JB9 capsid contains phospholipase A_2_ motifs—YxGxG (amino acids 285–289) and HdxxY (amino acids 308–312), required for parvovirus infectivity [17] and a glycine-rich region (amino acids 487–491) involved in parvovirus cell entry [18]. Seventy-four nucleotides upstream of the ORF1 start codon is a TATA box; the sequences in the 5′ region of the genome are predicted to form several small hairpins. Phylogenetic analysis (Fig. 1b) was performed using sequences of ORF1 and representative members of the *Copiparvovirus* genus and prototype strains of other *Parvovirinae* genera using MEGA 7 [19]. The analysis demonstrates the genetic relatedness of JB9 and MK1 with other members of the *Copiparvovirus* genus, in particular, BPV2 and the recently identified roe deer copiparvovirus which was originally detected in ticks raises some interesting possibilities in copiparvovirus transmission.

## Discussion

The identification of JB9 and MK1 as novel species in the *Copiparvovirus* genus adds to the growing number of parvoviruses identified in bovine serum [3, 6, 20]. To date, attempts to isolate the bovine parvovirus JB9 from the contaminated batch of serum in cell culture, including the inoculation of Madin–Darby bovine kidney cells, have been unsuccessful, possibly due the presence of neutralizing antibodies in the serum pool. Although the role of JB9 in bovine pathology, if any, remains to be elucidated, knowledge of the sequence and the availability of specific assays will be important for further investigations. Viruses detected in bovine serum pose a potential concern for manufacture of biological medicinal products when serum is used as a raw material or a medium supplement. This is similar to the detection of human parvovirus 4 (PARV4) in plasma fractionation pools and in factor VIII concentrates [21, 22]. Theiler’s disease, a form of equine hepatitis, has been reported to occur for over a century, albeit infrequently, in horses after the transfusion of different types of equine-derived blood products. The recent identification of EqPV-H, another copiparvovirus, and growing evidence of its role in Theiler’s disease [14] suggests that there may be further examples of copiparvovirus-associated pathologies which remain unexplored.

## Electronic supplementary material

Below is the link to the electronic supplementary material.

Supplementary material 1 (DOCX 56 kb)

## References

[1] Cotmore SF, Agbandje-McKenna M, Canuti M, Chiorini JA, Eis-Hubinger AM, Hughes J, Mietzsch M, Modha S, Ogliastro M, Pénzes JJ, Pintel DJ, Qiu J, Soderlund-Venermo M, Tattersall P, Tijssen P, ICTV Report Consortium (2019). ICTV virus taxonomy profile: parvoviridae. J Gen Virol.

[2] Li L, Giannitti F, Low J, Keyes C, Ullmann LS, Deng X, Aleman M, Pesavento PA, Pusterla N, Delwart E (2015). Exploring the virome of diseased horses. J Gen Virol.

[3] Sadeghi M, Kapusinszky B, Yugo DM, Phan TG, Deng X, Kanevsky I, Opriessnig T, Woolums AR, Hurley DJ, Meng XJ, Delwart E (2017). Virome of US bovine calf serum. Biologicals.

[4] de Souza WM, Dennis T, Fumagalli MJ, Araujo J, Sabino-Santos G, Maia FGM, Acrani GO, Carrasco AOT, Romeiro MF, Modha S, Vieira LC, Ometto T, Queiroz LH, Durigon EL, Nunes MRT, Figueiredo LTM, Gifford RJ (2018). Novel Parvoviruses from wild and domestic animals in brazil provide new insights into parvovirus distribution and diversity. Viruses.

[5] François S, Filloux D, Roumagnac P, Bigot D, Gayral P, Martin DP, Froissart R, Ogliastro M (2016). Discovery of parvovirus-related sequences in an unexpected broad range of animals. Sci Rep.

[6] Allander T, Emerson SU, Engle RE, Purcell RH, Bukh J (2001). A virus discovery method incorporating DNase treatment and its application to the identification of two bovine parvovirus species. Proc Natl Acad Sci USA.

[7] Cheung AK, Wu G, Wang D, Bayles DO, Lager KM, Vincent AL (2010). Identification and molecular cloning of a novel porcine parvovirus. Arch Virol.

[8] Xiao CT, Halbur PG, Opriessnig T (2013). Complete genome sequence of a novel porcine parvovirus (PPV) provisionally designated PPV5. Genome Announc.

[9] Ni J, Qiao C, Han X, Han T, Kang W, Zi Z, Cao Z, Zhai X, Cai X (2014). Identification and genomic characterization of a novel porcine parvovirus (PPV6) in China. Virol J.

[10] Phan TG, Gulland F, Simeone C, Deng X, Delwart E (2015). Sesavirus: prototype of a new parvovirus genus in feces of a sea lion. Virus Genes.

[11] Weber MN, Cibulski SP, Silveira S, Siqueira FM, Mósena ACS, da Silva MS, Olegário JC, Varela APM, Teixeira TF, Bianchi MV, Driemeier D, Pavarini SP, Mayer FQ, Roehe PM, Canal CW (2018). Evaluation of the serum virome in calves persistently infected with Pestivirus A, presenting or not presenting mucosal disease. Virus Genes.

[12] Linden A, Gilliaux G, Paternostre J, Benzarti E, Rivas JF, Desmecht D, Garigliany M (2019). A novel parvovirus, Roe deer copiparvovirus, identified in *Ixodes ricinus* ticks. Virus Genes.

[13] Divers TJ, Tennant BC, Kumar A, McDonough S, Cullen J, Bhuva N, Jain K, Chauhan LS, Scheel TKH, Lipkin WI, Laverack M, Trivedi S, Srinivasa S, Beard L, Rice CM, Burbelo PD, Renshaw RW, Dubovi E, Kapoor A (2018). New parvovirus associated with serum hepatitis in horses after inoculation of common biological product. Emerg Infect Dis.

[14] Tomlinson JE, Tennant BC, Struzyna A, Mrad D, Browne N, Whelchel D, Johnson PJ, Jamieson C, Löhr CV, Bildfell R, McKenzie EC, Laverack M, Renshaw RW, Dubovi E, Kapoor A, Meirs RS, Belgrave R, Engiles J, Van de Walle GR, Divers TJ (2019). Viral testing of 10 cases of Theiler's disease and 37 in-contact horses in the absence of equine biologic product administration: a prospective study (2014–2018). J Vet Intern Med.

[15] Zhang W, Li L, Deng X, Blümel J, Nübling CM, Hunfeld A, Baylis SA, Delwart E (2016). Viral nucleic acids in human plasma pools. Transfusion.

[16] Ignatov KB, Barsova EV, Fradkov AF, Blagodatskikh KA, Kramarova TV, Kramarov VM (2014). A strong strand displacement activity of thermostable DNA polymerase markedly improves the results of DNA amplification. Biotechniques.

[17] Zádori Z, Szelei J, Lacoste MC, Li Y, Gariépy S, Raymond P, Allaire M, Nabi IR, Tijssen P (2001). A viral phospholipase A2 is required for parvovirus infectivity. Dev Cell.

[18] Castellanos M, Pérez R, Rodríguez-Huete A, Grueso E, Almendral JM, Mateu MG (2013). A slender tract of glycine residues is required for translocation of the VP2 protein N-terminal domain through the parvovirus MVM capsid channel to initiate infection. Biochem J.

[19] Kumar S, Stecher G, Tamura K (2016). MEGA7: molecular evolutionary genetics analysis version 7.0 for bigger datasets. Mol Biol Evol.

[20] Gagnieur L, Cheval J, Gratigny M, Hébert C, Muth E, Dumarest M, Eloit M (2014). Unbiased analysis by high throughput sequencing of the viral diversity in fetal bovine serum and trypsin used in cell culture. Biologicals.

[21] Fryer JF, Kapoor A, Minor PD, Delwart E, Baylis SA (2006). Novel parvovirus and related variant in human plasma. Emerg Infect Dis.

[22] Fryer JF, Hubbard AR, Baylis SA (2007). Human parvovirus PARV4 in clotting factor VIII concentrates. Vox Sang.

