# Supplementary material for: Identification of a novel bovine copiparvovirus in pooled fetal bovine serum
Source: Virus Genes. 2020 Apr 18;56(4):522–6. doi: 10.1007/s11262-020-01757-1 (PMC7329774; doi:10.1007/s11262-020-01757-1)

**Identification of a novel bovine copiparvovirus in pooled fetal bovine serum**

Sally A. Baylis^1^, Csaba Miskey^1^, Johannes Blümel^1^, Marco Kaiser^2^, [Beatrix Kapusinszky](https://www.ncbi.nlm.nih.gov/pubmed/?term=Kapusinszky%20B%5BAuthor%5D&cauthor=true&cauthor_uid=22875894)^3^, Eric Delwart^3,4^

^1^Paul-Ehrlich-Institut, Langen, Germany

^2^GenExpress Gesellschaft für Proteindesign mbH, Berlin, Germany

^3^Vitalant Research Institute, San Francisco, California 94118, USA

^4^Department of Laboratory Medicine, University of California San Francisco, San Francisco, California 94118, USA

Corresponding author: Sally A. Baylis Sally.Baylis@pei.de

Journal: Virus Genes

**Keywords:** Parvovirus, *Copiparvovirus*, bovine sera

**Supplementary Materials and Methods**

**Sequence extension at the 5’ and 3’ ends of the virus genome**

To extend the sequence towards the 5’ end of the genome, a mutant Taq polymerase (SD polymerase) with high strand-displacement activity was used [16]. Ligating a linker to the resulting double stranded DNA end made it possible to amplify and sequence the 5’ end of the genome (Supplementary Fig. 1). Ten µl of DNA from the bovine serum was used in a 50 µl reaction containing 0.3 mM dNTPs, 3 mM MgCl_2_, 3 units of SD polymerase and enzyme reaction buffer (Bioron GmbH, Römerberg, Germany); the resulting mixture was incubated at 68°C for 5 minutes and product purified using Ampure XP magnetic beads (Beckman Coulter GmbH, Marburg, Germany) according to the manufacturer’s instructions and eluted in 20 µl of 0.1x TE buffer. The 5’ phosphorylation of the complete hairpin was performed with 100 units of T4 polynucleotide kinase (New England Biolabs GmbH, Frankfurt am Main, Germany) in T4 ligase buffer for 30minutes at 37°C in a 25 µl reaction volume. Ligation of double stranded linkers to the viral DNA was done by supplementing the reaction with 0.1 pmol of linker (5’-GTAATACGACTCACTATAGGGC) and 400 units of T4 DNA ligase and incubating the reaction at 4°C, overnight. Double stranded linkers were created by annealing the oligonucleotides TALinker-plus (5’-GTAATACGACTCACTATAGGGCTCCGCTTAAGGGACCATACGAGCTCTTCCGATCT) and TALinker-minus (5’-GATCGGAAGAGCTCGTATGA-Spacer) in 10 mM Tris HCl, 50 mM NaCl and 0.2 mM EDTA placed in a boiling water bath which was then switched off and allowed to cool. Five µl of the ligation reaction served as the template for the first round of PCR with 10 pmol of Linker-primer (5’-GTAATACGACTCACTATAGGGC) and parvo5 (5’-AGTTTACGCAGCCGTATGAA), using SD polymerase with the following cycling program: 92°C, 2 min; 30 cycles of 92°C 30 s, 55°C 30 s, 68°C 1 min; 68°C 5 min. After bead purification of the PCR product, one fifth of the reaction was used as a template for the nested PCR with Nested-primer (5’-AGGGCTCCGCTTAAGGGAC) and parvo5Nest (5’-GGCTCCTGTAGCTATACCTCT) using the PCR conditions described above. After agarose gel electrophoresis the PCR product was purified and Sanger sequencing was performed using the parvo5Nested primer.

To obtain further sequence at the 3’ end of the genome, PCR was performed using 1 pmol of a single primer biotinylated at the 5’ end (cont3Fbio 5’- AAAACACCACGAATGAAAGCC) using SD ploymerase and the following cycling program: 92°C, 2 min; 50 cycles of 92°C 30 s, 55°C 30 s, 68°C 1 min. The reaction was repeated with 0.5 µl of fresh SD polymerase. The biotinylated products were captured with Dynabeads M280 Streptavidin (Thermo Fisher Scientific, Darmstadt, Germany) at room temperature overnight. After washing the beads twice with water, the beads were resuspended in a 20 µl reaction volume of 0.2 µg random hexamers, 0.25 mM dNTPs, 1 U Klenow polymerase (New England Biolabs), in NEB2 buffer and incubated at 37°C for 50 minutes. After washing the beads with water, the free ends of the double stranded products were blunted and phosphorylated with the NEBNext End Repair Module and A-tailed using the NEBNext dA-Tailing Module (New England Biolabs). Ligation of the double-stranded linkers to the DNA ends and then nested PCRs were performed as described above with the following cycling conditions: 92°C 2 min; 30 cycles of 92°C 30 s, 55°C 30 s, 68°C 1 min; 68°C 5 min for the first PCR and 92°C 2 min; 30 cycles of 92°C 30 s, 60°C 30 s, 68°C 1 min; 68°C 5 min for the nested PCR. Primers for the first and second PCR were cont3Fbio and Linker-primer; and cont3F2 (5’- CCCAATGACACCTACACAACT) and Nested-primer, respectively. The resulting PCR products were cloned with the CloneJET PCR Cloning Kit (Thermo Fisher Scientific) and analysed by Sanger sequencing. An overview of the sequencing of the 5’ and 3’ regions of the viral genome is shown in Supplementary Fig. 1.

**Digital droplet PCR**

Nucleic acid extraction and ddPCR reactions were carried out as describer previously [15] using the following primers: C1-qPCR - Parvo C1 F 5’-GAGCCCGACACAAAGTCAGA-3’, Parvo C1 R 5’-TCCTCTTCTTCCTCTTCACCCT-3’ and Parvo C1 TM 5’-FAM-CCCAGTCATAGTCAGTCATGGAACGC- BBQ-3’. C2-qPCR – Parvo C2 F 5’-AAACTCACTGAAGTGGTGGGA-3’, Parvo C2 R 5’- GGCTGTCAGTCACTGTGGTG-3’ and Parvo C2 TM 5’-FAM-ATGTAACCTGCTCTGGCACAGAATCA-BBQ-3’; C3-qPCR – Parvo C3 F 5’- GCATCACAGCATACTAGACCAACT-3’, Parvo C3 R 5’-TTCTGACAAAGTTCACTTTAGGTATGA-3’ and Parvo C3 TM 5’-FAM-CACATTGCACTTGGAAATCACCCA-BBQ-3’. Copy number calculation, based on the Poisson distribution, was performed using the QuantaSoft software package (Bio-Rad, Munich, Germany).

**PCR analysis of bovine serum samples**

Samples were extracted using the QIAamp MinElute Virus Spin Kit (Qiagen, Hilden, Germany) with elution performed using 60 μl of elution buffer. Real-time PCR assays were performed using the three sets of primers used in the initial ddPCR analysis and performed using the LightCycler Multiplex DNA Master (Roche, Basel, Switzerland). Each 20 μl reaction mix contained 1× DNA-Master mix, 0.4 μM of the respective forward and reverse primers, 0.2 μM of each respective probe and 5 μl template DNA. Amplification and detection was performed using the LightCycler 480 Real-Time PCR System (Roche): 95°C for 5 min followed by 45 cycles at 95°C for 5 sec and 60°C for 15 sec and 72°C for 15 sec.

**Supplementary Table 1** Sequencing primers

| Primer code | Primer sequence | Sense |
| --- | --- | --- |
| ParV 329F | 5’-GACACTCGACGGAGAACCACA-3’ | S |
| ParV 740R* | 5’-CCCGAAGCACTCGTTGCA-3’ | A |
| ParV 668F | 5’-CGTAGGAGATCCGAGACACACA-3’ | S |
| ParV 1098R | 5’-GGTTTCCTGGTTGTGTGCA-3’ | A |
| ParV 1051F | 5’-ACTGCAGAGATGTCTACATGACCTATG-3’ | S |
| ParV 1525R | 5’-TTTGTTTGTTCTACAGAGGCTTCA-3’ | A |
| ParV 1473F | 5’-GTAGGCTGGTGGGAAGAAGG-3’ | S |
| Parvo 1875R (C3) | 5’-TTCTGACAAAGTTCACTTTAGGTATGA-3’ | A |
| Parvo 1784F (C3) | 5’-GCATCACAGCATACTAGACCAACT-3’ | S |
| ParV 2347R | 5’-CCCAGGGTCCATAGCTCA-3’ | A |
| ParV 2309F | 5’-CCATTGACGACCTCGAGCA-3’ | S |
| Parvo 2710R (C1) | 5’-TCCTCTTCTTCCTCTTCACCCT-3’ | A |
| Parvo 2613F (C1) | 5’-GAGCCCGACACAAAGTCAGA-3’ | S |
| Parvo 4140R (C2) | 5’-GGCTGTCAGTCACTGTGGTG-3’ | A |
| Parvo 4065F (C2) | 5’-AAACTCACTGAAGTGGTGGGA-3’ | S |
| ParV 4741R | 5’-CTTTGTGCAGTGATGCGTCTA-3’ | A |
| ParV 4664F | 5’-GCTACCCGCAAACAGACACA-3’ | S |
| ParV 5101R | 5’-AGTAGCCTGGAGCCTGTGGA-3’ | A |

S – sense; A – antisense; * sequencing primer for 5´-end sequencing; the number in the primer code refers to the position in the bovine parvovirus JB9 sequence.

The parvovirus specific conventional PCR assays were performed using the Q5 High-Fidelity DNA Polymerase (New England Biolabs GmbH, Frankfurt am Main, Germany). The 25µL reaction mixture contained DNA, 1x Q5 reaction buffer, 50 µM of each deoxynucleoside triphosphate dNTP, 0.5 µM forward and reverse primers and 1,25 U Q5 Polymerase. Amplification reactions were performed using an automated thermocycler (Eppendorf AG, Hamburg, Germany) with a hot start at 98°C for 2 min, followed by 45 cycles at 98°C for 30 sec, 64°C for 30 sec and 72°C for 30 sec and a final step at 72°C for 2 min. The PCR products were detected by agarose gel electrophoresis (staining gel with ethidium bromide, followed by visualization under UV light). For genome sequencing, PCR amplicons were extracted using the NucleoSpin Gel and PCR Clean-up kit (Macherey-Nagel GmbH & Co. KG, Düren, Germany). The recovered products were seqeunced using an ABI SeqStudio DNA Analyzer (Thermo Fisher, Darmstadt, Germany) using primers listed in Table 1. Sequences were assembled and manually edited to produce final sequences of the viral genome by MEGA 7 (http://megasoftware.net).

**Supplementary Fig. 1** Amplification of the 5’ and 3’ sequences of the bovine copiparvovirus 3 isolate JB9 genome.


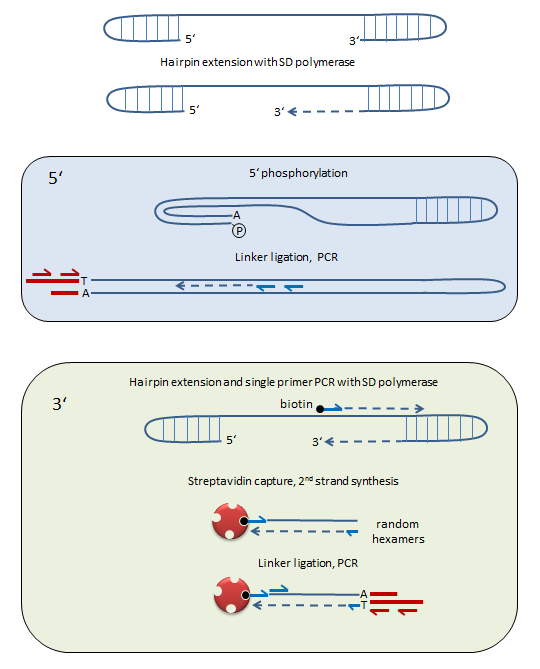

Supplement: Supplementary file 1 — Supplementary material 1 (DOCX 56 kb) [file 11262_2020_1757_MOESM1_ESM.docx]
